# Strengthening Triterpene Saponins Biosynthesis by Over-Expression of Farnesyl Pyrophosphate Synthase Gene and RNA Interference of Cycloartenol Synthase Gene in *Panax notoginseng* Cells

**DOI:** 10.3390/molecules22040581

**Published:** 2017-04-05

**Authors:** Yan Yang, Feng Ge, Ying Sun, Diqiu Liu, Chaoyin Chen

**Affiliations:** 1 Faculty of Life Science and Technology, Kunming University of Science and Technology, Kunming 650500, China; yangyan3650199@163.com (Y.Y.); zhengfanbj2008@163.com (Y.S.); dianaloveyou2006@163.com (D.L.); aqjingsheng@163.com (C.C.); 2 School of Biotechnology and Engineering, Dianxi Science and Technology Normal University, Lincang 677000, China

**Keywords:** farnesyl pyrophosphate synthase, cycloartenol synthase, *Panax*, over-expression, RNA interference, triterpene

## Abstract

To conform to the multiple regulations of triterpene biosynthesis, the gene encoding farnesyl pyrophosphate synthase (FPS) was transformed into *Panax notoginseng* (*P. notoginseng*) cells in which RNA interference (RNAi) of the cycloartenol synthase (CAS) gene had been accomplished. Transgenic cell lines showed both higher expression levels of *FPS* and lower expression levels of *CAS* compared to the wild-type (WT) cells. In the triterpene and phytosterol analysis, transgenic cell lines provided a higher accumulation of total triterpene saponins, and a lower amount of phytosterols in comparison with the WT cells. Compared with the cells in which RNAi of the *CAS* gene was achieved, the cells with simultaneously over-expressed *FPS* and silenced *CAS* showed higher triterpene contents. These results demonstrate that over-expression of *FPS* can break the rate-limiting reaction catalyzed by FPS in the triterpene saponins biosynthetic pathway; and inhibition of *CAS* expression can decrease the synthesis metabolic flux of the phytosterol branch. Thus, more precursors flow in the direction of triterpene synthesis, and ultimately promote the accumulation of *P. notoginseng* saponins. Meanwhile, silencing and over-expressing key enzyme genes simultaneously is more effective than just manipulating one gene in the regulation of saponin biosynthesis.

## 1. Introduction

*Panax notoginseng* is a well-known Chinese medicine herb. Triterpene saponins with a wide range of structural diversity are the major bioactive components in *P. notoginseng*. To date, over 70 different kinds of triterpene saponins have been isolated and characterized from different parts of *P. notoginseng* [1]. It has been proven that triterpene saponins have applications in anti-cancer [2], anti-atherosclerotic [3], anti-oxidant [4], anti-diabetic [5], anti-hypolipidemic [6] and some other pharmacological activities [7,8]. However, difficulties including a narrow habitat (mainly in Wenshan, China), long maturation period (>3 years) and crop rotation lead to a comparatively low production of *P. notoginseng*, and have restrained its pharmacological application. In addition, since the chemical structures of triterpene saponins are complicated, the chemical synthesis may give rise to high-cost. Therefore, production of triterpene saponins by metabolic engineering may be an appealing strategy to manipulate in the future.

**Figure 1:**
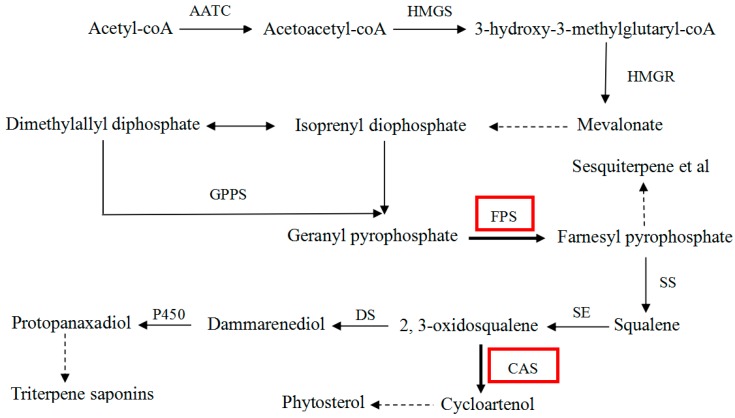
Biosynthetic pathway of triterpene saponins in *P. notoginseng*. AATC: acetoacetyl-CoA acyltransferase; DXPS: 1-deoxy-d-xyulose-5-phosphate synthase); HMGS: 3-Hydroxy-3-methylglutaryl-CoA synthase; DXR: 1-deoxy-d-xyulose-5-phosphate reductoisomerase; HMGR: 3-hydroxy-3-methylglutaryl-CoA reductase; CMS: 4-diphosphocytidyl-2-C-methyl-d-erythritol synthase; GPPS: geranyl pyrophosphate synthase; FPS: farnesyl pyrophosphate synthase; SS: squalene synthase; SE: squalene epoxidase; DS: dammarenediol-II synthase; P450: P450-monooxygenase; CAS: cycloartenol synthase. The dotted lines indicate several enzyme reactions.

The biosynthetic pathway of triterpene saponins in *P. notoginseng* is shown in Figure 1 [9]. FPS catalyzes the conversion of isoprenyl diophosphate (IPP) and dimethylallyl diphosphate (DMAPP) into farnesyl pyrophosphate (FPP) which acts as the common substrate in the biosynthesis of sesquiterpenoids, phytosterols and triterpene saponins. The conversion is a rate-determining reaction, therefore, this reaction catalyzed by FPS has been considered as the first pivotal step toward triterpene saponins and phytosterols biosynthesis [1]. Recently, the *FPS* gene has been cloned and characterized in some species [10,11], and research has suggested that the expression of *FPS* exhibits a positive correlation with triterpene saponin biosynthesis, and the accumulation of triterpene saponins can be up-regulated by the over-expression of *FPS* [12,13]. CAS in the pathway catalyzes the conversion of 2,3-oxidosqualene to cycloartenol which will be ultimately used to synthesize phytosterols. Although CAS does not participate in the biosynthesis of triterpene saponins, it competes with dammarenedion-II synthase (DS) for the same precursor (2,3-oxidosqualene). The 2,3-oxidosqualene is the common precursor of triterpene saponins and phytosterols. The catalysis of CAS may directly result in the decrease of 2,3-oxidosqualene and indirectly reduce the metabolic flux of triterpene saponin biosynthesis [14]. Thus, CAS should be considered as a key flux-limiting enzyme in the biosynthesis of triterpene saponins. In this study, the over-expression vector of *FPS* was constructed and integrated into the genome of *P. notoginseng* cells in which RNAi of *CAS* has been accomplished; such manipulation was used to confirm whether the strategy of multiple regulations was an effective way to strengthen the biosynthesis of triterpene saponins.

## 2. Results and Discussion

### 2.1. RNAi and Over-Expression Vector Construction

There are several methods to induce gene silencing in plants, such as anti-sense and co-suppression, but these constructs usually result in a modest proportion of silenced individuals. It has been reported that efficient gene silencing can be achieved by using hpRNA construct which contains a sense arm, anti-sense arm and an intron [15,16]. Based on these rules, the pHellsgate2 vector was designed to accept the *CAS* RNAi fragment (with attB1 and attB2 sites) via homologous recombination to form two arms of the hairpin and generate an ihpRNA construct.

**Figure 2:**
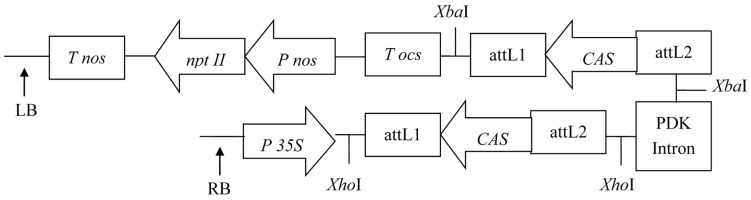
Schematic diagram of the pHellsgate-*CAS* RNAi vector. LB: left border; *P nos*: NOS promoter; *T nos*: NOS terminator; RB: right border. The *CAS* RNAi fragment was inserted into the *Xba*I sites and the *Xho*I sites of the pHellsgate2 by means of inverted repeats. After this step of the homologous recombination reaction, the hairpin structure (contains an antisense target sequence, the PDK intron and a sense target sequence) was inserted between the 35S promoter (*P 35S*) and OCS terminator (*T ocs*) of the pHellsgate2 vector, and fulfilled the function of gene silencing [15,16].

**Figure 3:**
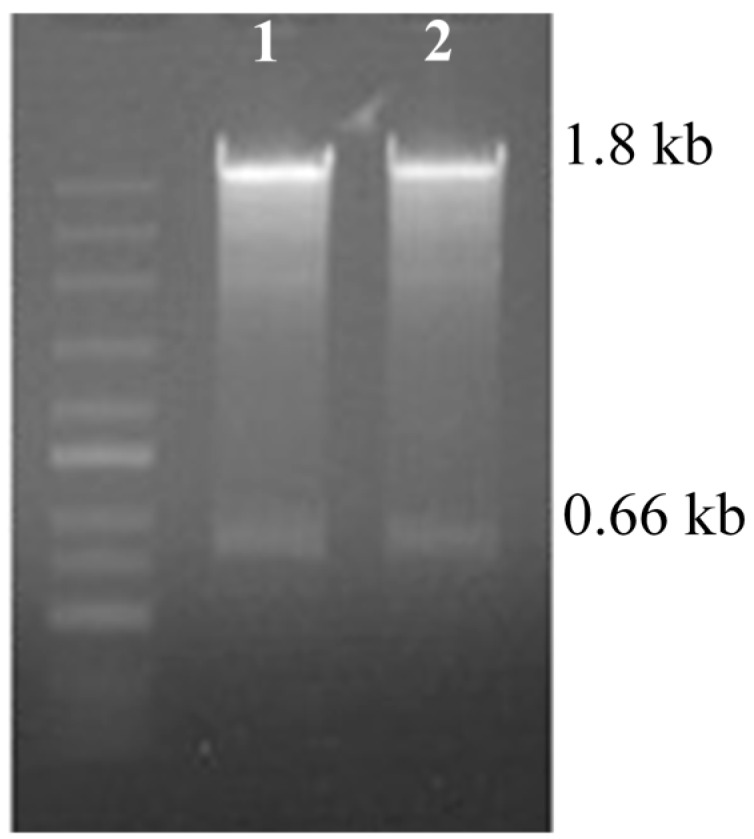
The enzymatic detection of pHellsgate-*CAS* by *Xba*I and *Xho*I. **1**: *Xba*I digestion products; **2**: *Xho*I digestion products.

Several members of a gene family can be simultaneously silenced by targeting the highly conserved sequence domain in the RNAi process. In order to guarantee the specificity of *CAS* silencing, the RNAi fragment was selected from the non-conservative region of the *CAS* sequence, and recombined into pHellsgate2 (Figure 2) to construct the RNAi expression vector pHellsgate-*CAS*. The pHellsgate-*CAS* was transferred into *E. coli* (*Escherichia coli*) DH5α. Positive clones were screened. The plasmid (pHellsgate-*CAS*) was isolated from the positive clone, and digested by *Xba*I and *Xho*I separately. Two fragments, 1.8 kb and 0.66 kb (the fragment of 0.66 kb contained 438 bp *CAS* RNAi fragments and there were 222 bp fragments between the enzyme digestion sites and recombination sites), were detected by agarose gel, and the size of the fragments digested by *Xba*I and *Xho*I was the same. The RNAi expression vector was constructed correctly (Figure 3).

The encoding region sequence of *FPS* (1033 bp) was amplified from the cDNA of *P. notoginseng* and successfully inserted into the pCAMBIA1300S vector.

### 2.2. Genetic Transformation of P. notoginseng

The cells cultured in vitro can be a useful system for genetic study and have been proven to possess many advantages, including high growth rate, genetic and biochemical stabilities and the totipotency of secondary metabolism [17]. Previous research has confirmed that cell cultures of *P. notoginseng* could increase the production of triterpene saponins [18,19]. In our study, on the base of callus cells, pHellsgate-*CAS* was transformed into the WT cells and pCAMBIA1300S-*FPS* was then introduced into the pHellsgate-*CAS* transformed cells. After 5~7 rounds of screening (each subculture period lasted about 4 weeks), the transgenic cell lines possessing kanamycin and hygromycin-resistance were obtained, and no morphological difference between transgenic and WT cells was observed.

### 2.3. PCR Analysis of Neomycin Phosphotransferase II (nptII) Gene and Hygromycin Phosphotransferase II (hptII) Gene

**Figure 4:**
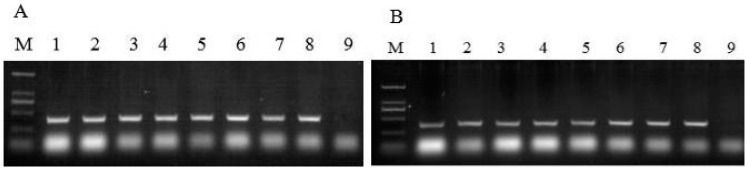
PCR detections of *nptII* and *hptII* in transgenic cell lines. (**A**) PCR detection of *nptII*; (**B**) PCR detection of *hptII*; M: *Trans2K^®^* DNA Marker; 1~7: Seven transgenic cell lines; 8: Positive control; 9: wild-type (WT).

Since the 438 bp fragment of *CAS* and the *nptII* possessed the same T-DNA, the integration of the exogenous 438 bp fragment of *CAS* in the genome of *P. notoginseng* cells could be confirmed by PCR analysis of *nptII*. Similarly, the *FPS* and the *hptII* possessed the same T-DNA, so the integration of exogenous *FPS* in the genome could be confirmed by PCR analysis of *hptII*. The genomic DNA of transgenic cell lines was isolated and used as the template for both *nptII* and *hptII* PCR. Both *nptII* and *hptII* fragments were detected in all transgenic cell lines, while no signal was observed in the WT cells (Figure 4A,B). This result indicated that the *CAS* RNAi fragment and *FPS* were simultaneously transformed into transgenic cell lines. With the exception of the two lines with poor growth, the other five PCR-positive cell lines (named T-1, T-2, T-3, T-4 and T-5) were used for further study.

### 2.4. Reverse-Transcriptase-PCR(RT-PCR) Analysis

**Figure 5:**
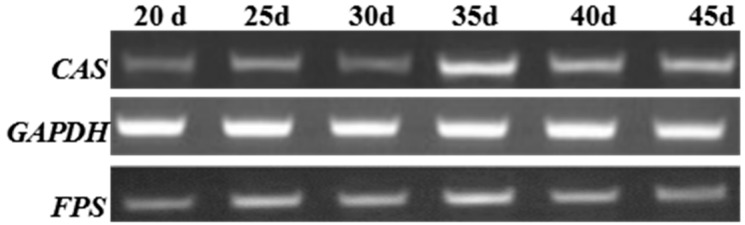
The expressions of *FPS* and *CAS* at different growth periods in *P. notoginseng* cells.

As the expressions of genes related to the biosynthesis of triterpene saponins in different growth periods were different, the expressions of *FPS* and *CAS* in different growth periods were examined by RT-PCR. The results showed that the expression levels of *FPS* and *CAS* on the 35th day were all higher than those in other growth periods (Figure 5). According to the result, the sampling time of the follow-up experiments was unified.

**Figure 6:**
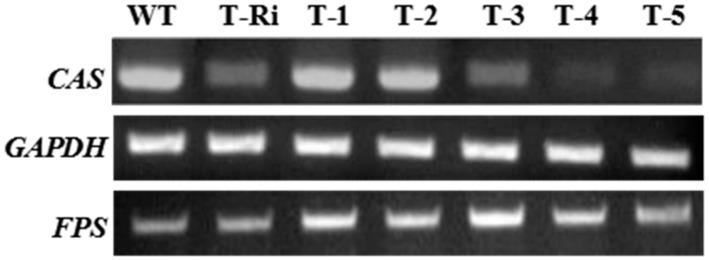
The RT-PCR results of *FPS* and *CAS* in *P. notoginseng* cells. T-Ri: cells transformed by the *CAS* RNAi fragment alone; T-1, T-2, T-3, T-4 and T-5: cells transformed by the *CAS* RNAi and *FPS* over-expression fragment simultaneously.

Among the five *nptII* and *hptII* PCR-positive transgenic cell lines, T-3, T-4, and T-5 showed both higher *FPS* and lower *CAS* transcription levels in comparison with the WT cells (Figure 6). The result demonstrated that the over-expression of *FPS* and *CAS* silencing were simultaneously accomplished in T-3, T-4, and T-5. Although the expression levels of *FPS* in T-1 and T-2 were increased, the expression levels of *CAS* did not show significant reduction. Possible reasons for this result may be (1) the insertion site of exogenous *FPS* into the genome happens to be a certain point in the *CAS* RNAi fragment, and the insertion of exogenous *FPS* blocks the expression of the *CAS* RNAi fragment, resulting in the ineffective inhibition of *CAS* expression; (2) during the screening process of transgenic *P. notoginseng* cells, genetic mutation of the RNAi fragment occurs in some cells, leading to the invalid expression of the RNAi fragment; (3) after several generations of continuous screening, the *CAS* RNAi fragment fell off the genome of transgenic cell lines.

Besides, compared with the cell line (T-Ri) which was only transformed by the *CAS* RNAi fragment, the transcription levels of *FPS* in T-3, T-4 and T-5 were all dramatically increased, and the expression of *CAS* showed a downward trend. This indicated that the expression of these two genes was not mutually exclusive in T-3, T-4 and T-5, and the three transgenic cell lines were then selected for further qPCR analysis.

### 2.5. qPCR Analysis

**Figure 7:**
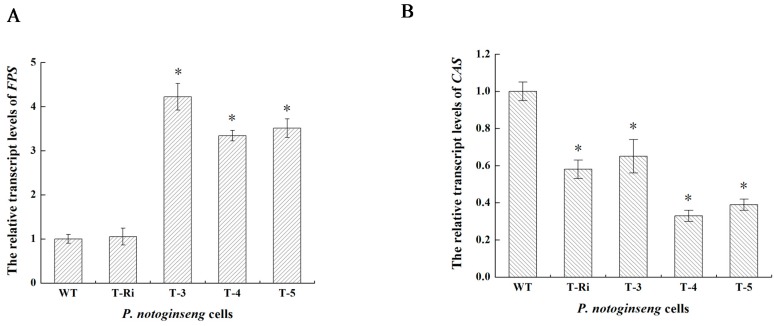
The relative transcription levels of *FPS* and *CAS* in *P. notoginseng* cells. (**A**) The relative transcription levels of *FPS*; (**B**) The relative transcription levels of *CAS*; The internal reference is *GAPDH*. Vertical bars indicate mean values ± standard deviations (SDs) from three independent experiments (*n* = 3). The symbol * represents that there is a significant difference between WT (control) and transgenic cells at *p* < 0.05.

To accurately verify the effects of *FPS* over-expression and *CAS* suppression, qPCR was carried out within the WT cells, T-Ri, T-3, T-4 and T-5 after cells had been cultured for 35 days. The results showed decreased accumulation of *CAS* transcript in T-Ri, T-3, T-4 and T-5 compared with the WT cells (0.58, 0.65, 0.33 and 0.39 folds of the WT cells, respectively). It confirmed again that the designed *CAS* RNAi fragment was efficient and could successfully inhibit the expression of *CAS* (Figure 7A). Meanwhile, the transcription levels of *FPS* in T-3, T-4 and T-5 all manifested an increasing trend compared with the WT cells (4.22, 3.34 and 3.51 folds of the WT cells, respectively), which demonstrated that over-expression of *FPS* in T-3, T-4 and T-5 had been realized (Figure 7B). Besides, in contrast to T-Ri in which only a single transgene was transformed, the decrease of *CAS* expression level in T-4 and T-5 was more significant. This suggested a possible interaction occurring between two transgenes, and the same phenomenon had been observed in the co-overexpression of *SS* and *DS* in *P. notoginseng* cells [20]. The detailed mechanism of such interaction should be investigated in the future.

### 2.6. Metabolic Analysis

**Figure 8:**
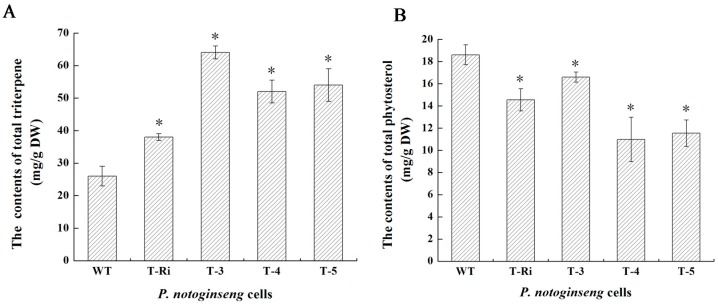
The contents of total triterpenes and phytosterols in *P. notoginseng* cells. (**A**) The contents of total triterpene; (**B**) The contents of phytosterol; Vertical bars indicate mean values ± SD from three independent experiments (*n* = 3). The symbol * represents that there is a significant difference between WT (control) and transgenic cells at *p* < 0.05.

**Figure 9:**
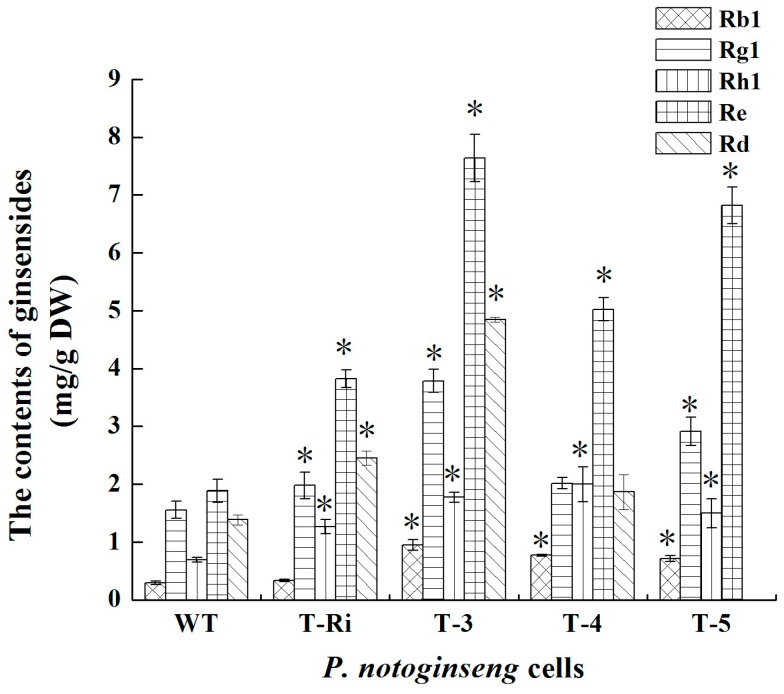
The contents of five major ginsenosides (Rb1, Rg1, Rh1, Re and Rd) in *P. notoginseng* cell lines. Vertical bars indicate mean values ± SD from three independent experiments (*n* = 3). The symbol * represents that there is a significant difference between WT (control) and transgenic cells at *p* < 0.05.

To investigate whether over-expression of *FPS* and silencing of *CAS* could influence triterpene and phytosterol biosynthesis, these metabolites from the WT, T-Ri, T-3, T-4 and T-5 cells were analyzed. The data showed that all transgenic cell lines (T-Ri, T-3, T-4 and T-5) produced significantly higher amounts of total triterpene saponins in comparison with the WT cells (1.46, 2.46, 2, 2.08 folds of the WT cells, respectively) (Figure 8A). It confirmed the hypothesis that the FPS in *P. notoginseng* was a key regulatory enzyme in the triterpene saponins biosynthetic pathway and its over-expression could raise the flux of the pathway and accordingly upregulated the accumulation of triterpene saponins. With the exception of the increased contents of saponins, phytosterols contents were reduced (0.77, 0.88, 0.58 and 0.61 folds of the WT cells, respectively) (Figure 8B). The result confirmed that *CAS* played a key role not only in the biosynthesis of phytosterols but also in the accumulation of triterpene saponins. The biosynthesis of triterpene saponins could be raised when the formation of phytosterols was blocked. This attractive conclusion was also confirmed in *Panax ginseng* hairy roots [14]. Meanwhile, HPLC analysis revealed that the contents of five major ginsenosides (Rb1, Rg1, Rh1, Re and Rd) in transgenic cell lines were all increased compared with those in the WT cells. Among the five ginsenosides, Re showed the highest rate of increase (the contents of Re in T-Ri, T-3, T-4 and T-5 were 2.03, 4.06, 2.66 and 3.62 folds of that in WT cells, respectively) (Figure 9). The monomer ginsenosides mentioned above are the main bioactive components which belong to tetracyclic triterpenoids and have been suggested to possess important pharmacological effects, such as neuroprotection activity (Rb1) [21,22], hypoglycemic activity (Rg1) [23], anti-tumor activity (Rh1) [24], antioxidant activity (Re) [25], and protective effects on the nervous system, immune system, and against cardiovascular and cerebrovascular diseases (Rd) [26]. In this study, the metabolic flux for triterpene skeleton formation was enhanced by the over-expression of *FPS* and the RNAi of *CAS*, which finally promoted the biosynthesis of tetracyclic triterpenoids. However, after the formation of the triterpene skeleton, different sorts of tetracyclic triterpenoids (Rb1, Rg1, Rh1, Re and Rd) would subsequently undergo different post-modification processes. It was related to a series of complex enzymatic actions during these processes, and consequently led to different increases of ginsenosides in transgenic cell lines. In addition, transgenic cell lines which were transformed with both the *CAS* RNAi fragment and *FPS* produced more triterpene saponins compared with the *CAS* RNAi fragment transgenic line (T-Ri). There was a synergistic effect among two transgenes in triterpene saponin biosynthesis. The multiple regulations including over-expressing key enzyme genes and blocking the by-product pathway were effective strategies to strengthen saponin biosynthesis in *P. notoginseng*.

Previous studies mainly focused on manipulating a single gene by means of over-expression or silencing, such as RNAi of *CAS*, thus enhancing the accumulation of triterpene saponins [14]. However, the amount of triterpene saponins produced by engineering a single gene is limited, so we suggest that manipulating two genes, governing the expression of rate-limiting enzymes, may result in a positive cooperative or synergistic effect to promote the accumulation of target compounds. Multigene transformation may be an appealing strategy to further enhance the triterpene saponin biosynthesis in some medicinal plants.

## 3. Materials and Methods

### 3.1. Plant Materials

*P. notoginseng* seeds were collected from Wenshan, Yunnan Province, China. The callus cells were induced from the seeds and cultured on Murashige and Skoog (MS) medium supplemented with 2 mg/L 2,4-dichlorophenoxyacetic acid (2,4-D), 1 mg/L kinetin (KT), 3% sucrose and 0.75% agar at 24 ± 1 °C in the dark. After continuous subculture, the callus cells in the vigorous growth period were employed as experimental materials during the whole research.

### 3.2. RNA Isolation and cDNA Synthesis

Total RNA was isolated from the fresh *P. notoginseng* cells by Tri-Reagent (MRC, Cincinnati, OH, USA) and the RNeasy plant Mini Kit (Qiagen, Duesseldorf, Germany) according to the manufacturer’s protocol. The integrality and concentration of total RNA samples were tested by using agarose gel electrophoresis and a UV/Visible spectrophotometer (GE, Fairfield, CT, USA), respectively. Then, 2 μg of total RNA was reverse transcribed with M-MLV reverse transcriptase (Promega, Madison, WI, USA). The obtained cDNA was used as a RT-PCR template to achieve the encoding region of *FPS* and the RNAi fragment of *CAS*.

### 3.3. RNAi Vector of CAS Construction

Suppression of *CAS* (GenBank accession number: EU342419) was achieved by the expression of a 438 bp RNAi fragment. The fragment was selected from the non-conservative region of *CAS* in order to guarantee the specific inhibition of *CAS* transcription. The RNAi fragment was amplified with a pair of specific primers, and the forward and reverse sequences of the primers were 5′-GGGGACAAGTTTGTACAAAAAAGCAGGCTGCGAGATGGTGGGTGGGGTTTG-3′ and 5′-GGGGACCACTTTGTACAAGAAAGCTGGGTCCACATAGGTTGCGTGCTTGATTC-3′, respectively. The PCR mixture was incubated in a 20 μL volume using a DNA thermal cycler (2720 thermal cycler, ABI, Foster City, CA, USA). The PCR conditions were as follows: 94 °C for 3 min; 32 cycles of 94 °C for 30 s, 57 °C for 30 s, 72 °C for 30 s; a final 5 min extension at 72 °C. After sequencing appraisal, the PCR products with the correct DNA sequences were inserted into the RNAi vector (pHellsgate2 which contained a *nptII* gene and conferred kanamycin resistance to plant cells) by a step of homologous recombination reaction mediated by Gateway^®^ BP Clonase™ II Enzyme Mix (Invitrogen, Carlsbad, CA, USA). The homologous recombination reaction was performed in a 10 μL volume containing 0.15 μg of PCR products, 0.15 μg of the pHellsgate2 vector, and 2 μL of BP Clonase™ II Enzyme Mix. After 4 h of incubation at 25 °C, the reaction was terminated by adding proteinase K and then incubated at 37 °C for 10 min. The recombinant vector pHellsgate-*CAS* was identified by enzyme digestion and subsequently transformed into *Agrobacterium tumefaciens* (*A. tumefaciens*) EHA105 competent cells by the freeze-thaw method [27].

### 3.4. Over-Expression Vector of FPS Construction

The complete encoding region of *FPS* (GenBank accession number: DQ059550.1) was amplified with a pair of specific primers carrying *Kpn*I and *Xba*I sites, respectively. The forward and reverse sequences of the primers were 5′-GGTACCAGAATGAGCGATCTGAAGACGAG-3′ and 5′-TCTAGAACAGACAACAACTTCCCCTCCAT-3′, respectively. The PCR mixture was incubated in a 20 μL volume, and the PCR conditions were as follows: 94 °C for 5 min; 32 cycles of 94 °C for 30 s, 58 °C for 30 s and 72 °C for 1 min 20 s; a final 15 min extension at 72 °C. After DNA sequence analysis, the PCR products with no mutations were inserted into pCAMBIA1300S which contained an *hptII* gene and conferred hygromycin resistance. The recombinant vector pCAMBIA1300S-*FPS* was then transformed into *A. tumefaciens* EHA105 competent cells via the above method [27].

### 3.5. Genetic Transformation of P. notoginseng Cells

The *A. tumefaciens* EHA105 harboring pHellsgate-*CAS* was firstly cultured at 28 °C for 3 days on solid LB medium with 50 mg/L kanamycin and 25 mg/L rifampicin. Then, the bacteria were collected with a vaccination needle and further cultured in liquid MGL medium containing 40 mg/L acetosyringone at 28 °C, 220 rpm/min until a final *A*_600_ between 0.6 and 0.8 was achieved. Meanwhile, the fresh wild-type (WT) cells were preincubated on MS solid medium with 40 mg/L acetosyringone, 2 mg/L 2,4-D, 1 mg/L KT at 25 °C for 3 days in darkness. Next, cells were collected and immersed in the *A. tumefaciens* suspension at 28 °C, 90 rpm/min for 25 min. After removing bacteria liquid, the cells were subsequently placed on MS solid medium with 40 mg/L acetosyringone, 2 mg/L 2,4-D, 1 mg/L KT and co-cultured at 25 °C for 3 days in darkness. Then, the cells were washed by sterile distilled water (repeated 3~4 times) and then incubated on MS solid medium with 400 mg/L cefotaxime, 2 mg/L 2,4-D, 1 mg/L KT for 15 days to inhibit the growth of EHA105. The final transformants with pHellsgate-*CAS* were selected on MS solid medium with 2 mg/L 2,4-D, 1 mg/L KT and 50 mg/L kanamycin. A total of 5~7 rounds of screening were conducted and each round lasted 35~50 days.

To obtain the cell lines harboring both pHellsgate-*CAS* and pCAMBIA1300S-*FPS*, the pCAMBIA1300S-*FPS* were secondly transformed into the kanamycin-resistant transformants according to the method described above except the selective MS solid medium containing 75 mg/L kanamycin, 30 mg/L hygromycin, 2 mg/L 2,4-D and 1 mg/L KT.

### 3.6. PCR Analysis of nptII and hptII Genes

Positive transgenic cell lines with both pHellsgate-*CAS* and pCAMBIA1300S-*FPS* were confirmed by genomic PCR analysis. Genomic DNA from transgenic cells and WT cells was isolated as described by Luo et al. [28]. The primers were designed to detect the 400 bp and 450 bp fragments of *nptII* and *hptII*, respectively, thus determining the presence of T-DNA in transgenic cells (Table 1). The PCR conditions of *nptII* and *hptII* were all performed as follows: 94 °C for 5 min; then 32 cycles of 94 °C for 30 s, 58 °C for 30 s, and 72 °C for 1 min; with a final 5 min extension at 72 °C. The PCR products were analyzed by agarose gel electrophoresis.

### 3.7. Semiquantitative RT-PCR Analysis

To unify the sampling time of the follow-up experiments, the expressions of *FPS* and *CAS* were analyzed in different growth periods. When the cells grew to 20 days, total RNA was extracted every 5 days for 45 days via the method described above. An amount of 2.0 μg of total RNA was reverse-transcribed by M-MLV Reverse Transcriptase (Promega, Madison, WI, USA). The primers used for RT-PCR are listed in Table 2. The *GAPDH* was used as a reference standard to normalize the expression level of each gene [29]. The PCR conditions were performed as follows: 94 °C for 5 min; then 32 cycles of 94 °C for 30 s, 58 °C for 30 s, and 72 °C for 30 s; with a final 5 min extension at 72 °C. The PCR products were analyzed by agarose gel electrophoresis. Thus, the best period of *FPS* and *CAS* expression was ascertained.

Next, the total RNAs from transgenic cell lines were isolated and used for further semi-quantitative RT-PCR analysis. According to the results, the transgenic cell lines with simultaneously enhanced *FPS* expression level and decreased *CAS* expression level were preliminarily screened.

### 3.8. Expression Analysis of FPS and CAS by Quantitative Real-Time PCR (qPCR)

Total RNAs from transgenic cells and WT cells were isolated at the best time of *FPS* and *CAS* expression. qPCR and RT-PCR shared the same gene-specific primers (Table 2). The qPCR was performed in a 20 μL volume containing 20 ng of cDNA, 10 μL of 2× GoTaq^®^ qPCR Master Mix (Promega, Madison, WI, USA), and 0.4 μL of 10 μM qPCR primers. The qPCR mixture was then programmed at 95 °C for 2 min, followed by 40 cycles of 95 °C 15 s, 60 °C for 30 s on a real-time system (ABI 7500, Foster City, CA, USA). The target quantity in each sample was normalized to the reference control (*GAPDH*) using the comparative method (2^−ΔΔCt^). Three independent experiments were performed.

### 3.9. Quantitative Analysis of Total Triterpene Saponins

The preparation of total triterpene saponins solution: WT cells, cells transformed by the *CAS* RNAi fragment (T-Ri) and cells with both *FPS* and the *CAS* RNAi fragment were collected and dried to the constant weight at 50 °C. Next, 0.5 g of these three kinds of dried cells were crumbled and transformed into a tube. After soaking in 50 mL 70%~75% methanol overnight, the cells were then treated with ultrasonic waves for 90 min (60 W, 4 s/5 s). Subsequently, the filtrate of methanol extract was collected and then evaporated. The dried residue was dissolved in 10 mL distilled water and then extracted with the same volume of water-saturated butanol 2~3 times. The residue was dissolved in methanol to constant volume of 25 mL after the solvent was evaporated. Finally, the evenly mixed liquid was filtered by 0.45 μm filter membrane.

The mixture of vanillin-perchloric acid as a coloring reagent was added to the total triterpene saponins solution, and the coloring reaction lasted 15 min at 60 °C. The absorbance at 550 nm of the reaction product was detected. According to the absorbance at 550 nm and standard curve, it was easy to obtain the content of the total triterpene saponins.

### 3.10. High-Performance Liquid Chromatography (HPLC) Analysis of Four Major Ginsenosides

The separation method of ginsennosides was described as follows: 0.1 g milled powder of dried cells was soaked in 70% methanol at 50 °C. After the liquid evaporated, the residue was dissolved in water and extracted with water-saturated butanol. The butanol layer was evaporated to produce a saponin fraction and dissolved in methanol. The determination of monomer ginsennoside content was carried out on an ULTIMATE 3000 LPG-3400A system (Dionex, Sunnyvale, CA, USA). The HPLC analysis was performed on a Waters Symmetry C18 column (5 μm, 4.6 mm × 250 mm) with water and acetonitrile as the mobile phase. Identification and quantification of ginsenosides were carried out by comparing their retention times and peak areas with those of standard ginsenosides purchased from National Institutes for Food and Drug Control (Beijing, China).

### 3.11. Quantitative Analysis of Totalphytosterols

The preparation of total phytosterols solution: WT cells, cells transformed by the *CAS* RNAi fragment (T-Ri) and cells with both *FPS* and the *CAS* RNAi fragment were collected and dried to the constant weight at 50 °C. Total phytosterols were extracted from 1.0 g of these three kinds of dried cells according to the alkali saponification method, and the details were elaborated by Liang [14].

The obtained purified total phytosterols were dissolved with 2 mL acetic anhydride at 60 °C. A drop of concentrated sulfuric acid was then added into the above solution, and the color of the mixture gradually changed from colorless to light green, and changed further from light green to dark green. The absorbance of the mixture with stabilized color at 650 nm was detected. According to the absorbance at 650 nm and standard curve, it was easy to obtain the content of the total phytosterols.

### 3.12. Statistical Analysis

The data were the averages of three independent sample measurements. The error bars indicated the standard deviations from the means of triplicates. The data were analyzed with the Student’s *t* test. The difference between WT (control) and transgenic cells was considered significant when *p* was <0.05 in a two-tailed analysis.

## 4. Conclusions

In this research, simultaneously over-expressing *FPS* and silencing *CAS* in *P. notoginseng* cells could regulate the expression levels of corresponding genes and thus enhance the accumulation of triterpenes. It was suggested that the over-expression of *FPS* could help to break the rate-limiting reaction catalyzed by FPS, thus providing sufficient precursors for triterpene biosynthesis. The inhibition of *CAS* expression decreased the synthesis metabolic flux of the phytosterol branch with allowing more precursors to flow in the direction of triterpene synthesis, and ultimately promoting saponin production. Furthermore, compared with the interference of a single gene, the simultaneous interference and over-expression of double genes led to further improvement of triterpene biosynthesis. This study lays good foundations for the future commercial large-scale production of triterpene saponins via a multi-gene transformation system in *P. notoginseng* cells.

## Figures and Tables

**Table 1:** Primer sequences used for genomic PCR.

Gene	Forward Primer Sequence (5′–3′)	Reverse Primer Sequence (5′–3′)
*nptII*	CTCTGATGCCGCCGTGTT	CCCTGATGCTCTTCGTCCA
*hptII*	GAAGTGCTTGACATTGGGGAAT	AGATGTTGGCGACCTCGTATT

**Table 2:** Primer sequences of *CAS*, *FPS* and *GAPDH*.

Gene	Forward Primer Sequence (5′–3′)	Reverse Primer Sequence (5′–3′)
*FPS*	CGGATGCTGGACTATAATGTG	ATTTACGGCAATCATACCAACC
*CAS*	CGGATGGTTTATCTGCCTATGTC	GGTTGCGTGCTTGATTCCA
*GAPDH*	CTACCAACTGTCTTGCTCCCCT	TGATGCAGCTCTTCCACCTCTC
